# Effects of Local and Systemic Metronidazole as Adjunctive Treatment in Chronic Periodontitis Patients

**DOI:** 10.1002/cre2.70050

**Published:** 2024-12-01

**Authors:** Maryam Mehravani, Ehsan Houshyar, Sheida Jamalnia, Rasool Gharaaghaji

**Affiliations:** ^1^ Student Research Committee Qazvin University of Medical Sciences Qazvin Iran; ^2^ Department of Periodontics, Faculty of Dentistry Urmia University of Medical Sciences Urmia Iran; ^3^ Department of Nursing and Midwifery, Kazeroun Branch Islamic Azad University Kazeroun Iran; ^4^ Student Research Committee Shiraz University of Medical Sciences Shiraz Iran; ^5^ Department of Biostatistics, School of Medicine Urmia University of Medical Sciences Urmia Iran

**Keywords:** adjunctive therapy, antibiotic, chronic periodontitis, metronidazole

## Abstract

**Objectives:**

This study aimed to compare the effects of local and systemic metronidazole in patients with chronic periodontitis.

**Materials and Methods:**

In this randomized clinical study, 30 patients (3 teeth per patient) were treated in three groups: scaling and root planing (SRP) treatment alone, metronidazole tablet as adjunctive treatment, and metronidazole gel as adjunctive treatment. BOP (bleeding on probing), PPD (pocket probing depth), and CAL (clinical attachment level) data were collected at the beginning and 3 months later. Collected data were tested by Wilcoxon and Kruskal–Wallis tests.

**Results:**

BOP, CAL, and PPD levels were significantly different at the beginning of treatment and after 3 months, and this was true for all treatments. BOP, CAL, and PPD levels did not differ significantly between the three groups after the treatment (*p* > 0.05).

**Conclusion:**

The effectiveness of these methods was all equal and SRP is still considered as the gold standard in the treatment of periodontal diseases. Further studies are needed to confirm the findings. Chronic periodontitis is a progressive disease that can cause tooth loss. The accepted treatment is SRP. Antibiotics used systemically can penetrate the depth of the periodontal pockets but have several side effects. Hence, using a less complicated medicinal form as a topical gel as adjunctive therapy in treating chronic periodontitis can be more effective.

**Trial Registration**: Iranian clinical trial https://en.irct.ir/: IRCT20210408050898N1

## 1 Introduction

Chronic periodontitis is an inflammatory and progressive disease causing the destruction and loss of tooth‐supporting tissues (Klokkevold, Newman, and Takei 2015a). Periodontal diseases are highly prevalent and affect about 20%–50% of the world population (Nazir 2017). Various bacteria, such as Gram‐negative species of obligate anaerobes, are critical factors in periodontal diseases (Klokkevold, Newman, and Takei 2015b). Accordingly, successful treatment of this disease depends on stopping tissue destruction and eliminating or controlling etiological factors. Currently, the accepted treatment protocol is regular scaling and root planning (SRP) in affected areas. Nevertheless, pathogenic organisms may remain in periodontal tissues and dentinal tubules that cannot be removed by mechanical treatment alone (Greenstein and Caton 1990).

Thus, due to the association of periodontal disease with anaerobic microorganisms, antimicrobial regimens have been added to conventional mechanical methods in treating this disease (Klokkevold, Newman, and Takei 2015c). Several studies have evaluated antimicrobial agents such as metronidazole, amoxicillin, azithromycin, tetracycline, doxycycline, and clindamycin and their effects on treating invasive and chronic periodontitis (Luchian et al. 2021). Antibiotics used systemically penetrate the tooth's periodontal tissues and deep into the periodontal pockets. Therefore, systemic antibiotics can also target bacteria unreachable by mechanical debridement techniques or local antibiotics (Slots and Ting 2002). On the other hand, long‐term systemic antibiotic therapy increases the risk of problems such as antibiotic resistance (Loesche 1996) and adverse drug reactions such as nausea, diarrhea, and pseudomembranous colitis (Slots and Ting 2002). In addition, systemic use of antibiotics requires high patient cooperation. Due to such problems, studies focusing on developing local drug systems to release antibiotics in periodontal pockets are increasing (Rams and Slots 1996). Various drug delivery systems such as gels, micro and nanoparticles, fibers, and strips have been available recently (Jepsen and Jepsen 2016). These systems increase the drug concentration at the target site and minimize the side effects of systemic drug use (Rams and Slots 1996). Due to the similar clinical results of different antibiotics, none is superior to the others (Luchian et al. 2021). Metronidazole is effective against periodontal pathogens and bactericidal against anaerobic organisms and is usually used alone or combined with amoxicillin in periodontitis treatment (Manoor). Various studies have demonstrated the clinical benefits of metronidazole (gel or tab) in treating chronic periodontitis (Slots and Ting 2002; Loesche 1996; Rams and Slots 1996; Manoor; Loesche et al. 1992, 1991; Kadkhoda et al. 2012). Hence, in the present study, metronidazole (used as a local gel or as a systemic tablet) as an adjunct to conventional mechanical therapy (SRP) by the ultrasonic device in patients with chronic periodontitis referred to Alborz dentistry clinic and dental office in Urmia, Iran, was evaluated.

## 2 Materials and Methods

This study is a single‐blind, randomized clinical trial to compare the effects of metronidazole gel and tablets (in intervention groups) with the SRP alone (in the control group).

### 2.1 Study Population

The study population consisted of patients beyond Stage II periodontitis (localized or generalized) and Grade B referred to the Alborz dentistry clinic and dental office in Urmia, Iran. Based on the results of the Kadkhoda et al. (2012) study, considering α = 0.05 and power of 0.8 and using the formula for calculating the sample size for the mean difference in the population, the number of samples was 28. Including shedding, the sample size was 30 patients in each study group selected by random sampling method. Inclusion criteria included those over 30 years of age, patients diagnosed with beyond Stage II periodontitis (localized or generalized) and Grade B, with at least three pockets with probing depth greater than 4 mm, good general health, patients who have not had any anti‐inflammatory drugs in the last 6 months or have not taken antibiotics, patients who have at least 15 teeth (except the third molar) in their mouths, patients who have good cooperation, and who have signed an informed written consent form. Patients without inclusion criteria and an O'Leary plaque index of more than 15% after proper oral health education and a history of allergy to metronidazole were excluded from the study.

### 2.2 Randomization

In this study, 30 patients (3 teeth per patient) diagnosed with beyond Stage II periodontitis (localized or generalized) and in Grade B were divided into a 10‐person control group and two 10‐person intervention groups based on a pre‐generated randomization order, which was random, and the arrangement pattern was completely random. The block randomization method with 3 and 6 blocks was used to divide the samples.

### 2.3 Intervention

The researcher was blind to the type of drug prescribed to each patient. Informed consent was obtained from the patients, and all stages of the study were explained. All patient information remained confidential. The ethics code was obtained from the ethics committee of Urmia University of Medical Sciences: IR.UMSU.REC.1399.319, and the trial was registered in Iranian clinical trials (https://en.irct.ir/) IRCT20210408050898N1.

**Figure 1:**
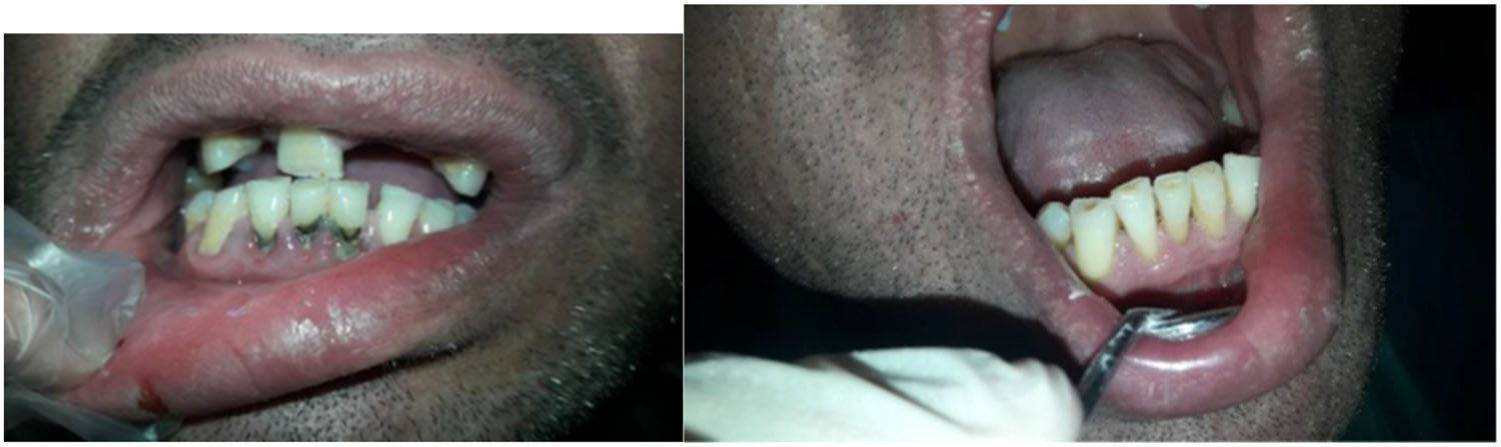
Before and after standard SRP treatment alone by the ultrasonic device.

**Figure 2:**
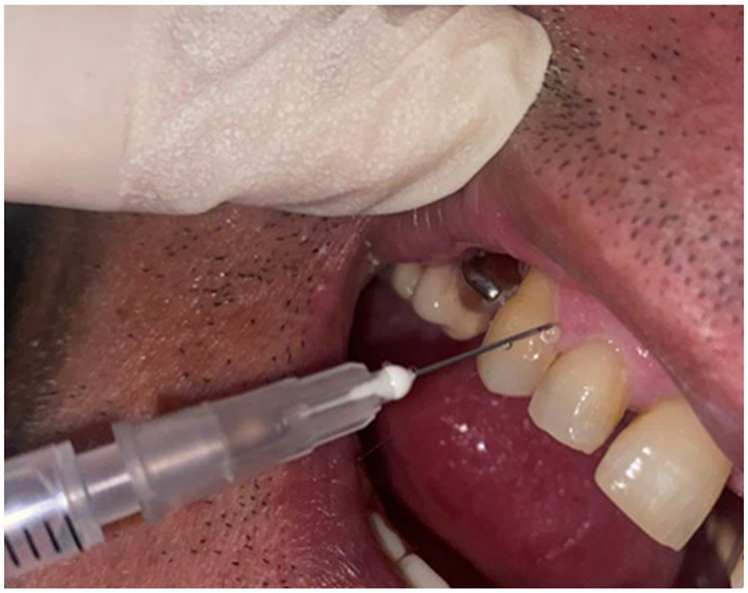
Metronidazole gel insertion.

Patients in the control group (Group A) received only standard SRP treatment by the ultrasonic device (Figure 1). Also, all patients in intervention groups first received standard SRP treatment. Patients in intervention Group B received adjuvant therapy in the form of metronidazole tablets (250 mg every 8 h for 7 days) by a second person (Noyan et al. 1997). In patients in intervention Group C after standard SRP, 0.75% metronidazole gel (Parseh, Tehran, Iran) was administered inside three pockets in the study by the second person using a syringe with a non‐cutting end until the pocket was completely filled (Figure 2). Patients were advised not to eat or drink for 2 h after receiving the gel (Noyan et al. 1997). The gel was reused in the same way 1 week, 2 weeks, and 3 weeks later by the same person (Kadkhoda et al. 2012). The plaque control of patients included in the study was evaluated every 2 weeks by assessing the O'Leary plaque index.

Mean pocket probing depth (PPD), mean clinical attachment loss (CAL), and bleeding on probing (BOP) in randomly selected pockets equal to or greater than 4 mm in each patient were evaluated by a periodontal probe (Probe UNC 15; Hu‐Friedy, Chicago, IL, USA) at two times, including:

1. Before starting the treatment.
2. 3 months after starting the treatment.

### 2.4 Data Analysis

First, central tendency and dispersion measures were calculated and described for each group.

So, the Shapiro‐Wilk test was used to check the normality of the variables. Then Wilcoxon test was used to evaluate different treatments' effects on BOP, CAL, and PPD at the beginning of treatment and after 3 months and to compare the mean of the variables in several groups, we used the Kruskal–Wallis test. Statistical analysis was performed using SPSS Statics for Windows, version 22.0. Armonk, NY, USA: IBM Corp; 2013; the significant level was set at *p* < 0.05.

## 3 Results

There were 12 females (40%) and 18 males (60%) out of 30 participants. The results for all CAL, PPD, and BOP parameters at the beginning of treatment (Index i) and after 3 months (Index f) for all treatment groups were less than 0.0001, revealing all data have skewed distribution.

Table 1 shows the mean of BOP, PPD, and CAL parameters in the three treatment groups. This table shows the changes in these parameters for the three treatment groups before treatment and after 3 months. The Kruskal–Wallis test was used to compare the effect of drug use in the three groups. BOP had the most significant reduction in treatment with SRP alone and the group receiving metronidazole gel as an adjunctive treatment. The mean CAL in the side treatment with metronidazole gel and the mean PPD in the side treatment with metronidazole tablets or gel had the highest decrease.

**Table 1:** Comparison of changes in measured parameters between the three treatment groups.

Parameter	Study group	Primary	Final	Mean decrease
BOP (%)	MTZ gel	0.87	0.20	0.67
	MTZ tablet	0.73	0.20	0.53
	SRP	0.77	0.10	0.67
CAL (mm)	MTZ gel	5.83	4.67	1.16
	MTZ tablet	4.97	3.88	1.09
	SRP	4.78	4.07	0.71
PPD (mm)	MTZ gel	4.90	3.83	1.07
	MTZ tablet	4.70	3.65	1.05
	SRP	4.32	3.60	0.72

Since the data had a skewed distribution, the Wilcoxon test was used to evaluate different treatments' effects on BOP, CAL, and PPD, and the results are shown in Table 2. Based on the results, the values of BOP, CAL, and PPD at the beginning of treatment (Index i) and after 3 months (Index f) are significantly different, and this is true for all treatments.

**Table 2:** The results of the Wilcoxon test to evaluate the effect of different treatment methods on the measured parameters.^a^

Parameter	Group								
	MTZ gel			SRP			MTZ Sys.		
	BOP_f_ BOP_i_	CAL_f_ CAL_i_	PPD_f_ PPD_i_	BOP_f_ BOP_i_	CAL_f_ CAL_i_	PPD_f_ PPD_i_	BOP_f_ BOP_i_	CAL_f_ CAL_i_	PPD_f_ PPD_i_
*p* value	< 0.001	< 0.001	< 0.001	< 0.001	< 0.001	< 0.001	< 0.001	< 0.001	< 0.001

^a^ f: after three months; i: at the beginning of the treatment.

The Kruskal–Wallis test was performed to evaluate and compare the effects of the type of treatment in three groups, and the results are shown in Table 3.

**Table 3:** Results of the Kruskal–Wallis test to examine the relationship between treatment groups.^a^

Variable	PPD_f_	CAL_f_	BOP_f_	PPD_i_	CAL_i_	BOP_i_
P value	0.922	0.236	0.491	0.080	0.139	0.424

^a^ f: after three months; i: at the beginning of the treatment.

At the beginning of treatment (with Index i) and after 3 months (with Index f), the values of BOP, CAL, and PPD did not differ significantly between the three groups (*p* > 0.05). In other words, all treatments have been equally effective.

## 4 Discussion

Anaerobic bacteria are one of the critical factors in periodontal diseases (Klokkevold, Newman, and Takei 2015b). Since SRP treatment, which is the standard treatment in periodontal diseases, is not able to remove all the bacteria in the depth of the pocket (Greenstein and Caton 1990), local or systemic antimicrobials can access the deeper parts of periodontal pockets.

According to the said contents and considering that currently, the use of local drug therapy systems is worthy of attention, the purpose of this one‐sided blind study is to investigate the effect of local 0.75% metronidazole gel as an adjunctive treatment in the treatment of patients suffering from beyond Stage II periodontitis in the Department of Periodontology, Faculty of Dentistry, Urmia University, Urmia, Iran.

Since the use of systemic metronidazole as an adjunctive treatment in the treatment of periodontal diseases has been recommended in numerous articles (Slots and Ting 2002; Loesche 1996; Jepsen and Jepsen 2016; Loesche et al. 1992; Noyan et al. 1997; Faveri, Figueiredo, and Feres 2014; Sgolastra et al. 2021), the use of 0.75% metronidazole gel can be considered as an alternative to systemic metronidazole only when it has at least the same effect as systemic metronidazole.

In this one‐sided blind study, patients diagnosed with periodontitis beyond Stage II and Grade B were classified into three groups receiving SRP treatment, SRP treatment plus systemic metronidazole as an adjunctive treatment, and SRP treatment plus metronidazole gel as an adjunctive treatment.

All study subjects received SRP treatment because antimicrobial substances have little effect on biofilms, and mechanical debridement leads to an increased impact of the drug, locally and systemically (Pandit et al. 2013).

A calibrated clinician, utterly blinded to the type of treatments in each of the groups, evaluated the clinical parameters. This blinding was done to reduce the potential of bias.

Metronidazole gel was renewed every 7 days in the gel group because of its short substantivity to have an adequate level of the drug in the envelopes (Pandit et al. 2013). In addition, it has been shown that the experience of the operator performing the mechanical debridement and the time spent for SRP are essential factors in the effectiveness of the treatment, and the lack of adequate debridement in the pockets can lead to treatment‐resistant pockets (Perinetti et al. 2004). For this reason, in this study, all SRPs were completed by an experienced operator without setting a time limit to perform SRP in one or, if necessary, two sessions to minimize the confounding effects of not having a proper SRP. Because of the different levels of plaque and calculus in different patients, each patient may need to spend less or more time. In this study, the patients' plaque index was checked every 2 weeks to eliminate the effect of inappropriate plaque control on the treatment results and to assess the cooperation of the patients in complying with oral hygiene education. So, patients with more than 15% plaque index were excluded from the study due to the confounding effects of plaque and the high probability of patient non‐cooperation.

The ideal recall interval in the treatment of supportive periodontal diseases is 90 days (Perinetti et al. 2004), which was considered in this study.

In this study, BOP, PPD, and CAL were significantly different at the beginning of treatment and after 3 months, and this is true for all three treatments. In other words, there was a significant difference in PPD levels at both stages of the study (*p* < 0.001). Furthermore, PPD levels and BOP did not differ significantly between the three groups at the beginning of treatment and after 3 months. In other words, all treatments were equally effective, and no significant difference was seen (*p* > 0.05). Nevertheless, in a randomized clinical trial in 2011, Kadkhoda et al. (2012) evaluated the effect of local antibiotic therapy with metronidazole with SRP in treating aggressive periodontitis. Statistical comparison of PPD between the control and study group showed that in 6–12 weeks of follow‐up after the initial treatment, the *p*‐value reached from 0.58 (insignificant) to 0.002 (significant) and then 0.001 (significant). Also, the mean BOP at the beginning of treatment was almost the same between the two groups, but over time, there was a more significant decrease in the experimental group than the control group, making a statistically significant difference between the two groups (*p* = 0.005). Also, in 2019, Montaruli et al. (2019) conducted a clinical trial study in Italy to evaluate the effect of local metronidazole with nonsurgical treatment in patients with chronic periodontitis. They used the Wilcoxon test for PPD values before and after treatment in the control and experimental groups, which significantly changed patients' treatment response (*p *< 0.05). Some reasons for these differences are the relatively small number of studies, the low power of the study to detect statistical disparities, and the low time considered for the analysis of clinical parameters. In the reviewed articles, in some cases, it was seen that the clinical parameters were not different at 1–3 months, and then at 3–12 months, these parameters were significantly different. Also, in most studies on local metronidazole usage, higher concentrations of metronidazole gel have been used. Our study used a concentration of 0.75% due to the lack of access to higher concentrations. Similar to the present study, Bergamaschi et al. (2016) conducted a pilot clinical trial to compare the effect of local metronidazole gel or systemic tablet use as adjunctive therapy in line with complete oral debridement in smoker patients with chronic periodontitis. PPD improved in all groups from the first month to the last evaluation time (*p *< 0.05). However, no statistically significant difference (*p* > 0.05) was observed between the groups when each time point was considered separately. Also, in Perinetti's study regarding the effect of 1% metronidazole gel as an adjunctive treatment, the changes in the mean BOP, PPD, and CAL between the group receiving the gel and the control group, despite the evidence of clinical improvement, were not statistically different from each other. Pandit et al. (2013) evaluated the effect of minocycline microparticles and 25% metronidazole gel as an adjunct therapy with SRP in treating chronic periodontitis in a randomized clinical trial. In this study, patients receiving SRP treatment with metronidazole gel had a more clinically significant improvement in CAL than the control group. Nevertheless, the difference in CAL increase was not statistically significant. In recent years, various methods and drugs have been used to treat periodontal diseases.

Anti‐rheumatoid medications are among the drugs intended for the treatment of periodontal diseases. Considering the inflammatory nature of both rheumatoid arthritis and periodontal diseases, various drugs with anti‐inflammatory and immune system‐modulating properties that are used in the treatment of rheumatoid arthritis have also been investigated in periodontal therapy, such as MMP (matrix metalloproteinases) inhibitors, anticytocins, corticosteroids, DMARDs (disease‐modifying antirheumatic drugs). However, the definitive effect of these drugs on the treatment of periodontal disease is unknown and requires more studies in this field (Martu et al. 2021).

One of the other interesting methods studied by Nicolae et al. (2015) is the use of photoactivated blue‐O Toluidine in treating peri‐implantitis. Peri‐implantitis is a condition in which pathogens on the implant lead to a series of inflammatory responses in the periodontium surrounding the implant. Apparently, in peri‐implantitis, we have an increase in those bacterial species that have an essential role in periodontitis. In this study, the intervention group with peri‐implantitis was treated with Photoactivated Blue‐O Toluidine in addition to SRP, and the results indicated that patients in the intervention group showed significant improvement in pocket depth and local inflammation parameters.

Other treatments that have received attention in recent years are natural substances for oral and dental care and periodontal health maintenance. The reason for this attention is the more natural, available, low cost, and high safety of these compounds. For example, it has been shown that various substances such as *Anacardium occidentale* extract, Soy isoflavones, and other bioflavonoids, dentifrice containing *S. persica*, dentifrice containing *Carica papaya*, citratus, and *A. indica* extract have shown remarkable results in improving various periodontal parameters (Budala et al. 2023).

## 5 Limitations

The findings of this study have to be seen in light of some limitations. At first, we can mention the relatively small number of investigated cases. Due to the coincidence of this study with the COVID‐19 pandemic and the lack of access to the calculated sample size, three teeth from each person were included in the study. As a result, the study may not have enough power to detect statistical differences.

The second limitation is the 3‐month follow‐up time to check the clinical parameters. In the reviewed articles, in some cases, it was seen that the clinical parameters were without significant difference between 1 and 3 months, and after that, between 3 and 12 months, these parameters have found a significant difference. Also, in most of the studies regarding the use of topical medicine, higher concentrations of metronidazole gel have been used. In this study, due to existing sanctions and lack of access to metronidazole gel with higher concentrations, the concentration of 0.75% has been used to evaluate the effectiveness of this accessible concentration. Therefore, to confirm these findings, more studies with a larger statistical population, more extended follow‐up periods, or higher concentrations of metronidazole gel, if available, are needed.

## 6 Conclusion

Metronidazole gel and tablet and SRP treatment were all effective in improving the clinical parameters of CAL, PPD, and BOP. Metronidazole locally or systemically in patients with periodontitis beyond Stage II and Grade B compared to SRP alone did not affect clinical findings. And SRP treatment is still considered as the gold standard in the treatment of periodontal diseases. Further studies are needed to confirm the results.

## Author Contributions

Maryam Mehravani and Ehsan Houshyar convinced the original idea and carried out the experiment and also wrote the manuscript with the support of Sheida Jamalnia and Rasul Ghareaghaji.

## Ethics Statement

All the interventions performed in this study, which included human participants, conformed to the ethical standards of the Institutional and National Research Committee and following the 1964 Declaration of Helsinki and its subsequent amendments, as well as ethical standards.

## Consent

Written informed consent was obtained from all patients participating in the study. All patient information remained confidential.

## Conflicts of Interest

The authors declare no conflicts of interest.

## Data Availability

The data that support the findings of this study are available on reasonable request from the corresponding author. The data are not publicly available due to privacy or ethical restrictions. Pseudonymous data is available from the corresponding author upon reasonable request.
